# Survival model database of human digestive system cells exposed to electroporation pulses: An *in vitro* and *in silico* study

**DOI:** 10.3389/fpubh.2022.948562

**Published:** 2022-09-05

**Authors:** Xuan Han, Nana Zhang, Yuchi Zhang, Zhuoqun Li, Yingxue Wang, Lujing Mao, Tianshuai He, Qingshan Li, Jiawen Zhao, Xue Chen, Yixuan Li, Zitong Qin, Yi Lv, Fenggang Ren

**Affiliations:** ^1^Department of Hepatobiliary Surgery, The First Affiliated Hospital of Xi'an Jiaotong University, Xi'an, China; ^2^National Local Joint Engineering Research Center for Precision Surgery and Regenerative Medicine, The First Affiliated Hospital of Xi'an Jiaotong University, Xi'an, China; ^3^Institute of Regenerative and Reconstructive Medicine, Med-X Institute, The First Affiliated Hospital of Xi'an Jiaotong University, Xi'an, China

**Keywords:** electroporation pulses, irreversible electroporation, digestive system, numerical analysis, mathematical model

## Abstract

**Background and objectives:**

This study aimed to establish a mathematical survival model database containing cell-specific coefficients from human digestive system cells exposed to electroporation pulses (EPs).

**Materials and methods:**

A total of 20 types of human digestive system cell lines were selected to investigate the effect of EPs on cell viability. Cell viability was measured after exposure to various pulse settings, and a cell survival model was established using the Peleg–Fermi model. Next, the cell-specific coefficients of each cell line were determined.

**Results:**

Cell viability tended to decrease when exposed to stronger electric field strength (EFS), longer pulse duration, and more pulse number, but the decreasing tendency varied among different cell lines. When exposed to a lower EFS (<1,000 V/cm), only a slight decrease in cell viability occurred. All cell lines showed a similar tendency: the extent of electrical injury (EI) increased with the increase in pulse number and duration. However, there existed differences in heat sensitivity among organs.

**Conclusions:**

This database can be used for the application of electroporation-based treatment (EBT) in the digestive system to predict cell survival and tissue injury distribution during the treatment.

## Introduction

Induced by electroporation pulses (EPs), pulsed electric fields (PEF) can alter the cell transmembrane potential and cause nanometer-sized defects or pores on the cell membrane, leading to reversible electroporation (RE) or irreversible electroporation (IRE) (1). In terms of RE, EPs can induce transitory exchange channels for exogenous molecules or substances into the cytoplasm and maintain cell viability (2); in terms of IRE, EPs can eliminate cancer or diseased tissue *in situ* through electrical injury (EI) without thermal injury (TI) (3, 4). As EPs can physically modulate cell membrane permeability and cause a series of alterations in cellular physiological activity, electroporation-based treatment (EBT) is considered a unique physical method for biotechnology, cancer treatment, and tissue ablation (3).

Although the mechanism of cell electroporation is still controversial, it is well-established that the electric field distribution plays a major role in the biological outcome of EPs, which is primarily determined by the pulse parameter, electrode configuration, environmental conditions, and tissue properties (5, 6). Therefore, the intensity and biological effects of EBT are theoretically controllable and predictable by applying the proper parameters to specific tissues. However, the parameter settings in EBT is often empirical and estimative, which may lead to undesired results, such as unexpected cell death when performing cell electrotransfection or unexpected tumor residue in tumor ablation (7, 8). Thus, it is necessary to provide mathematical evidence for the EBT to apply proper pulse settings. In 2014, Dermol and Miklavčič have demonstrated that mathematical models of cell permeabilization and survival can be used for clear treatment planning and better tissue damage prediction (9).

Electroporation is not a simple or instantaneous process but a cumulative effect based on pulse intensity and quantity. The pulse voltage, duration, number, and frequency should be considered to estimate EP outcomes. By combining *in vitro* experiment data and mathematical modeling, Dermol and Miklavčič compared the goodness of fit and applicable situation among different mathematical models of electroporation, confirming Peleg-Fermi model is suitable for treatment planning of electrochemotherapy and IRE (10). Therefore, the Peleg–Fermi model, which includes the parameters of electric field strength (EFS), pulse number, pulse duration, and several cell-specific coefficients, has been established to estimate the biological effect of the electroporation process with prominent efficacy in calculating the EI (9, 11). The cell-specific coefficient in the Peleg–Fermi model is a series of parameters reflecting the tendency and extent of cell viability changes exposed to EPs of a certain pulse duration and frequency, which may differ between different organs and even different cells. However, although the thermal effect is not indispensable for the electroporation process, there is still the possibility of thermal effect and even TI during the EPs when applying a high voltage to the cell or tissue due to the tissue resistance of electrical properties, which should be avoided during EBT (12, 13). The thermal effect is primarily determined by the temperature distribution, which can be calculated using Penne's bioheat transfer equation and the Arrhenius equation (14). The Joule heat generated by the EPs was incorporated into Pennes' bioheat transfer equation as an external heat source and increased the tissue temperature through time-dependent and electrical-thermal coupling effects that were determined by the pulse duration, number, and frequency (15, 16). Based on the mathematical model, it is possible to achieve a non-thermal EBT by adjusting the pulse parameter and electrode configuration for a specific tissue. Therefore, it is necessary to further optimize the mathematical model and investigate the specific parameters for various tissues, which is meaningful for the further application of EBT.

However, there is only one series of cell-specific coefficient data available for prostate cancer (17), rendering it inaccurate in model simulation and treatment planning for disease of other organs (18). As mentioned above, the pulse parameters are crucial for the outcomes of EBT; thus, it is reasonable to set the proper pulse parameter to achieve the expected results. Various types of EBT have been successfully used in cancer treatment and tissue ablation, such as electrochemotherapy (19), pulsed field ablation (20), and IRE. Moreover, the feasibility, safety, and efficacy of EBT for the focal treatment of cavity organs, which are usually the restricted areas of thermal-based ablation techniques, have been investigated. In our previous studies, the safety and efficacy of IRE in the stomach and bile duct have been investigated with favorable outcomes (21–24). These results demonstrate that EBT is an attractive candidate for the treatment of such endoluminal tumors. However, there is a lack of cell-specific coefficient data from the digestive system that renders it inaccurate for model simulation and treatment planning.

Herein, a mathematical model database containing the cell-specific coefficient from 20 types of human digestive system cell lines (including the esophagus, stomach, colon, liver, bile duct, and pancreas) exposed to EPs was established by *in vitro* and *in silico* studies. In addition, the efficacy of the survival model for IRE was evaluated via numerical analysis of the digestive system.

## Materials and methods

### Cell lines and cell culture

A total of 20 types of human digestive system cell lines, including 17 cancer cell lines and three normal cell lines, were selected to investigate the effect of EPs on cell viability *in vitro*. Detailed information on the cell lines is provided in Table 1. Major cells lines were chosen to be accord with primary pathological type of digestive tumor (e.g., squamous carcinoma in esophagus). All cells were cultured in Dulbecco's Modified Eagle Medium (DMEM; Biological Industries Co., Ltd., Kibbutz Beit-Haemek, Israel) supplemented with 10% fetal bovine serum (Biological Industries Co., Ltd., Kibbutz Beit-Haemek, Israel) and 1% penicillin–streptomycin solution (Beyotime Biotechnology, Nantong, Jiangsu, China). All cultures were maintained in a 5% CO_2_ humidified incubator (Thermo Forma 371, Thermo Fisher Scientific, Waltham, MA, USA) at 37°C.

**Table 1:** The information for the cell lines.

**Organ**	**Cell line**
Esophagus	KYSE-150, KYSE-410
Stomach	GES-1, MGC-823, SGC-7901, MKN-45
Colon	SW-620, SW-480, HCT-116, LoVo
Bile duct	HIBEpiC, HuCCT-1, QBC-939, HCCC-9810
Liver	L-02, Huh-7, Hep-3B, Hep-G2
Pancreas	PANC-1, MIA PaCa-2

### EPs protocols

Before exposure to EPs, the cells were washed with phosphate-buffered saline solution, harvested by trypsin, and resuspended in DMEM culture medium to a concentration of 8 × 10^4^ cells/ml. The cell suspension (500 μl) was added to a 2 mm gap cuvette (45-0135, Harvard Apparatus, Holliston, MA, USA). A square wave pulse generator (BTX ECM 830, Harvard Apparatus, Holliston, MA, USA) was used to generate a burst of EPs. The detailed protocol of the pulse setting for each procedure is summarized in Supplementary Table 1.

### Cell counting kit-8 experiment for cell viability

After exposure to EPs, the cell suspension was re-collected immediately, dispensed into 96-well plates, and incubated for 24 h. Cell viability was assessed using the cell counting kit (CCK)-8 commercial kit (Dojindo Molecular Technologies Inc., Kumamoto, Japan) following the manufacturer's instructions. The optical density (OD) value at a wavelength of 450 nm was measured using an automatic microplate reader (Varioskan Flash Multiplate Reader, Thermo Fisher Scientific, Waltham, MA, USA). Cell viability was calculated using the following equation:


(1) $$\begin{array}{c} S=\frac{Aexp-Ablan}{Acon-Ablan}\times 100\% \end{array}$$

where *Aexp, Ablan*, and *Acon* are the mean OD values of the experimental, blank medium, and control groups, respectively. For each parameter setting of each cell line, the experiment was repeated 10 times. All data were analyzed using GraphPad (9.0, GraphPad Software Inc., San Diego, CA, USA).

### Curve fitting for mathematical model

The equation for the Peleg–Fermi model was as following:


(2) $$\begin{array}{ccc} S & = & \frac{1}{1+e^{\frac{E-E_{c}\left(N\right)}{A_{c}\left(N\right)}}} \end{array}$$

(3) $$\begin{array}{ccc} E_{c}\left(N\right) & = & E_{0}\exp\left(-k_{1}N\right) \end{array}$$

(4) $$\begin{array}{ccc} A_{c}\left(N\right) & = & A_{0}\exp\left(-k_{2}N\right) \end{array}$$

where *S* is the cell viability after EP; *E* is the EFS; *E*_*c*_*(N)* and *A*_*c*_*(N)* are two functions that depend on the pulse number (*N*) and cell-specific coefficients, where *E*_*c*_*(N)* represents the EFS required for the half lethality of cells, and *A*_*c*_*(N)* is the constant of the cell survival curve slope under specific pulse parameters; and *E*_0_*, A*_0_*, k*_1_, and *k*_2_ are the coefficients related to the pulse duration, frequency, and cell type. *R*^2^ was calculated as statistical measure for the goodness of fit, which indicates the correlation between the calculated and practically determined value. The value of *E*_*c*_*(N)* and *A*_*c*_*(N)* was calculated based on Equation (2) as first step. Then the value of the coefficients was calculated subsequently based on the results of the first step and Equations (3) and (4), respectively. Thus, two *R*^2^ values was acquired in step 2 because *E*_0_ and *k*_1_ were fitted separately from *A*_0_ and *k*_2_ based on different equation.

Although the efficacy of the Peleg–Fermi model has been demonstrated by previous studies, it is still possible that the data of a certain cell line from the *in vitro* study were not suitable for curve fitting. To solve this problem, the modified Peleg–Fermi model and power function model was used instead (18, 25, 26).

The equation for the modified Peleg–Fermi model was as following:


(5) $$\begin{array}{ccc} E_{c}\left(N\right) & = & E_{1}exp\left(-k_{1}N\right)+E_{2}exp\left(-k_{2}N\right) \end{array}$$

(6) $$\begin{array}{ccc} A_{c}\left(N\right) & = & A_{1}exp\left(-k_{3}N\right)+A_{2}exp\left(-k_{4}N\right) \end{array}$$

where *E*_1_*, E*_2_*, A*_1_*, A*_2_*, k*_1_*, k*_2_*, k*_3_, and *k*_4_ are the coefficients related to the pulse duration, frequency, and cell type.

The equation for the power function model was as following:


(7) $$\begin{array}{c} E=a{\left(\frac{t_{p}}{t_{0}}\right)}^{b} \end{array}$$

where *t*_*p*_ is the pulse duration given in (ms) and *t*_0_ = 1 ms.

The survival model curve fitting and calculation of the coefficients were referenced from Goldberg et al. (27), and were performed using MATLAB software (R2020b, The Mathworks Inc., Natick, Massachusetts, USA). The MATLAB program code is shown in the Supplementary materials. The values of *E*_*c*_*(N)* (*N* = 100) were estimated using Equation (3) for each cell line.

In Pelge-Fermi equation, with the increase of the pulse number, even very weak electric filed will induce electric injury, which is inconsistent with the reality. Therefore, a threshold is needed to limit the calculation for IRE. Here, we defined *E*_*c*_*(N)*, the field at which 50% of a population of cells, as the threshold for subsequent numerical analysis.

### Numerical analysis for evaluation

To evaluate the efficacy of the mathematical model for IRE, a numerical analysis was performed using COMSOL Multiphysics (Version 5.6, COMSOL Co., Ltd., Burlington, MA, USA) using the finite element method in accordance with our previous study (16). To simplify the calculation, a two-dimensional tissue model with a couple of needle electrodes were established. The tissue domain was isotropic and homogeneous. The electrical and thermal properties of the tissue (including the esophagus, stomach, colon, liver, bile duct, and pancreas) are obtained from an open database and summarized in Table 2 (28). Detailed information on the simulation model is described in Supplementary materials.

**Table 2:** The electrical and thermal properties of tissues.

**Property**	**Esophagus**	**Stomach**	**Conlon**	**Bile duct**	**Liver**	**Pancreas**	**Electrode**
Electrical conductivity (S/m)	0.511	0.511	0.012	0.900	0.028	0.511	1.000 × 10^6^
Density (kg/m3)	1040.000	1088.000	1088.000	1070.500	1078.750	1086.500	2700.000
Heat capacity (J/kg°C)	3500.000	3690.000	3655.000	3716.000	3540.200	3164.000	1000.000
Thermal conductivity (W/m°C)	0.530	0.525	0.540	0.521	0.519	0.512	250.000
Blood perfusion rate (ml/min·kg)	190.000	460.320	765.000	30.000	860.500	767.500	N/A
Heat generation rate (W/kg)	2.940	7.130	11.850	0.465	9.931	11.886	N/A

A time-dependent study was performed to investigate the accumulative electrical and thermal effects of EP on the tissue. Specifically, the pulse parameter was set to 2,000 V in pulse voltage, 100 μs in pulse duration, and 1 Hz in frequency. A total of 100 pulses were used for EI analysis. To fully evaluate the thermal effect of IRE and heat sensitivity of each organ, 200 pulses (equal to 200 s) were set for the analysis of TI.

Because the results from the Peleg–Fermi model and Arrhenius model depicted survival rate, the threshold of irreversible injury for the probability of EI and TI was set as 0.5, implying that if the probability of EI or TI was >0.5, it could be treated as an irreversible injury. The effective electric field was defined as the threshold of EFS for electroporation that was set as the corresponding value of *E*_*c*_*(N)* for each cell line.

To quantitatively evaluate the model from a certain cell line, the relative area ratio of the probability of EI and the probability of TI were calculated using Equations (7) and (8):


(8) $$\begin{array}{ccc} \text{Relative area ratio of EI} & = & S_{EI}/S_{total} \end{array}$$

(9) $$\begin{array}{ccc} \text{Relative area ratio of TI} & = & S_{TI}/S_{total} \end{array}$$

where *S*_*EI*_ is the area of EI, *S*_*TI*_ is the area of TI, and *S*_*total*_ is the total area of the tissue domain. All data were analyzed and compared using GraphPad (9.0, GraphPad Software Inc., San Diego, CA, USA). As the calculation of EI is based on the specific coefficients obtained from the Peleg–Fermi model at the cellular level, the corresponding *S*_*EI*_ can be acquired for each of the 20 cell lines. Since the calculation of TI is based on the Arrhenius equation at the organ level, *S*_*TI*_ can only be obtained for each organ.

### Statistical analysis

Statistical analysis was performed using GraphPad. The results are expressed as mean ± SD.

## Results

### *In vitro* assessment of viability of cells exposed to EPs

**Figure 1:**
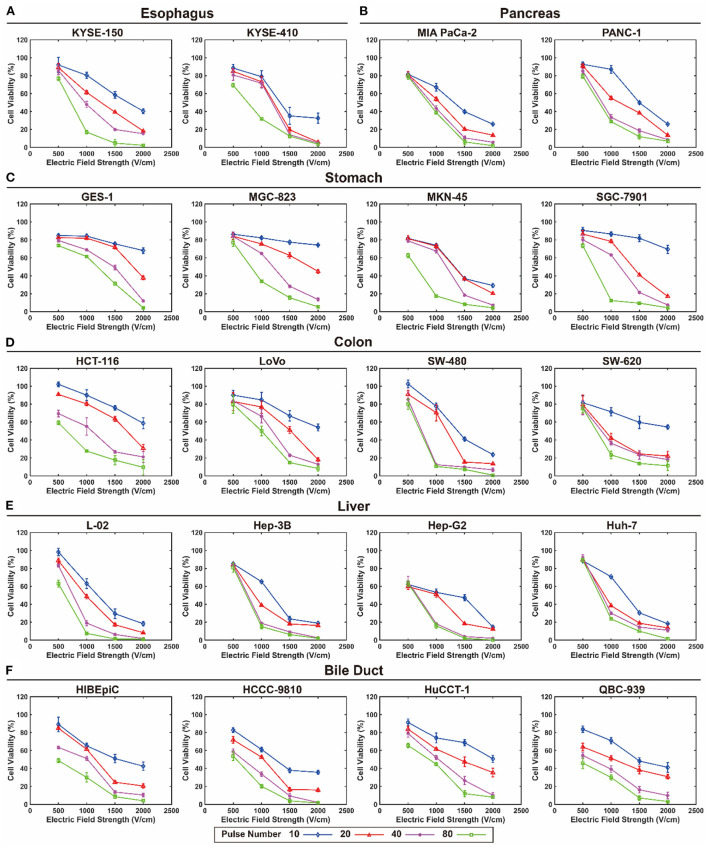
Viability of each cell line exposed to electroporation pulses with 100 μs pulse duration under various pulse numbers and electric field strengths. **(A)** Esophagus, **(B)** pancreas, **(C)** stomach, **(D)** colon, **(E)** liver, and **(F)** bile duct.

The viability of each cell line exposed to EPs under various pulse durations, pulse numbers, and EFS was determined. Figure 1 shows the viability of each cell line exposed to EPs with a 100 μs pulse duration under various pulse numbers and EFS. The detailed data are summarized in the online database (29). Generally, cell viability tended to decrease with an increase in EFS, pulse duration, and pulse number, but the decreasing tendency was different among different cell lines. However, when exposed to a lower EFS (500 V/cm), only a slight decrease in cell viability occurred (Figure 1). In contrast, when exposed to a short pulse duration, cell viability even increased under a lower EFS.

### Survival curve based on the mathematical model

**Figure 2:**
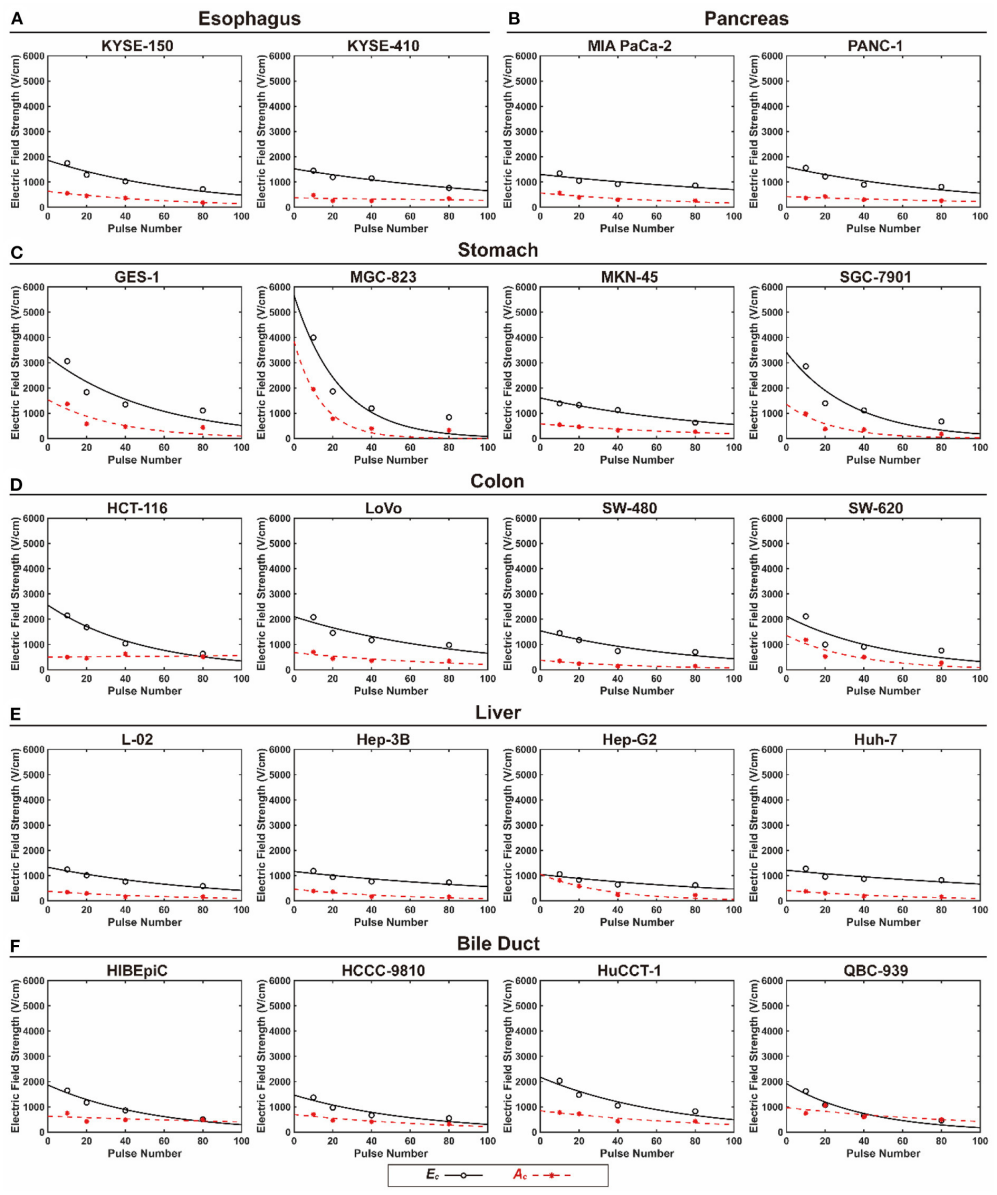
The fitting curve of *E*_*c*_ and *A*_*c*_ for each cell lines with the increase in pulse number under 100 μs pulse duration. **(A)** Esophagus, **(B)** pancreas, **(C)** stomach, **(D)** colon, **(E)** liver, and **(F)** bile duct.

Table 3 summarizes the cell-specific coefficients, *E*_*c*_, *A*_*c*_, and *E*_*c*_ *(100)* of the 20 cell lines under different pulse durations. Figure 2 shows the fitting curve of *E*_*c*_ and *A*_*c*_ for each cell line with the increase in pulse number under a pulse duration of 100 μs. The data of *E*_*c*_ and *A*_*c*_ under pulse durations of 25, 50, and 75 μs are available in the online database. Moreover, the results of power function model of all cell lines are available in the online database as well (29). The lethality of the EPs increases with the accumulation of pulses, leading to a decline in *the E*_*c*_ and *A*_*c*_ values. Furthermore, the decline rate of *the E*_*c*_ and *A*_*c*_ values varied among the cells (Figure 2).

**Table 3:** The cell specific coefficients of the 20 digestive system cell lines under different pulse duration.

**Organ**	**Cell line**	**Pulse duration (μs)**	***E_0_* (V/cm)**	***k_1_***	***R* ^2^**	***A_0_* (V/cm)**	***k_2_***	***R* ^2^**	*E_*c*_(100)* (V/cm)*
Esophagus	KYSE-150	25	2756.213	0.010	0.885	681.381	0.005	0.628	1013.954
		50	2714.890	0.013	0.950	1088.084	0.016	0.863	739.894
		75	2059.141	0.014	0.963	644.249	0.014	0.926	507.778
		100	1862.259	0.014	0.961	634.689	0.015	0.995	459.227
	KYSE-410	25	2173.894	0.009	0.922	333.371	0.003	0.215	883.839
		50	2001.763	0.010	0.915	362.119	0.013	0.957	736.407
		75	1785.091	0.010	0.997	176.637	0.000	0.024	656.698
		100	1524.046	0.008	0.968	372.925	0.003	0.258	684.798
Stomach	GSE-1	25	3690.107	0.010	0.955	646.374	−0.001	0.182	1357.515
		50	3444.518	0.015	0.944	959.728	0.010	0.871	768.576
		75	3510.741	0.018	0.920	1210.089	0.022	0.837	580.322
		100	3244.770	0.018	0.894	1533.835	0.028	0.823	536.357
	MGC-823	25	4539.967	0.016	0.939	980.346	0.007	0.766	916.604
		50	3546.443	0.015	0.939	1172.765	0.013	0.942	791.318
		75	3651.689	0.020	0.966	1268.881	0.017	0.963	494.202
		100	5669.169	0.042	0.945	3843.981	0.071	0.978	85.012
	MKN-45	25	3082.787	0.010	0.961	963.341	0.011	0.893	1134.094
		50	2492.628	0.010	0.932	1011.575	0.018	0.951	916.987
		75	2171.824	0.013	0.996	723.632	0.014	0.989	591.891
		100	1605.015	0.011	0.982	582.408	0.011	0.962	534.263
	SGC-7901	25	3686.389	0.016	0.975	960.052	0.016	0.938	744.269
		50	2990.836	0.015	0.944	832.338	0.014	0.915	667.346
		75	3271.241	0.025	0.954	993.818	0.025	0.937	268.520
		100	3407.636	0.029	0.915	1355.732	0.042	0.905	187.499
Colon	SW-620	25	2386.836	0.008	0.942	740.177	0.009	0.949	1072.475
		50	2243.286	0.009	0.908	720.728	0.004	0.569	912.052
		75	2140.876	0.010	0.936	806.931	0.005	0.897	787.584
		100	2116.665	0.019	0.800	1354.309	0.028	0.882	316.587
	SW-480	25	2296.339	0.010	0.995	569.940	0.005	0.660	844.776
		50	2161.404	0.010	0.967	890.061	0.008	0.877	795.136
		75	1677.443	0.012	0.891	523.802	0.007	0.985	505.236
		100	1542.091	0.013	0.915	377.732	0.018	0.884	420.269
	HCT-116	25	2699.715	0.010	0.927	264.336	−0.004	0.401	993.170
		50	3305.015	0.018	0.964	546.569	0.002	0.224	546.315
		75	2560.682	0.016	0.944	319.794	−0.007	0.924	516.993
		100	2556.739	0.020	0.990	503.801	−0.001	0.228	346.017
	LoVo	25	2678.650	0.009	0.964	491.367	0.009	0.977	1089.058
		50	2481.962	0.009	0.930	720.808	0.014	0.925	1009.090
		75	2417.015	0.012	0.905	645.304	0.017	0.783	727.991
		100	2100.307	0.012	0.901	675.382	0.012	0.805	632.600
Liver	L-02	25	2193.061	0.007	0.980	375.922	0.001	0.092	1089.042
		50	1539.792	0.007	0.956	430.113	0.006	0.940	764.638
		75	1271.158	0.007	0.878	468.985	0.019	0.916	631.238
		100	1332.386	0.012	0.972	379.581	0.014	0.911	401.307
	Hep-3B	25	2139.251	0.011	0.946	343.131	0.002	0.354	712.095
		50	1576.574	0.009	0.921	426.943	0.010	0.938	640.987
		75	1430.396	0.008	0.854	428.361	0.014	0.891	642.718
		100	1165.961	0.007	0.871	463.969	0.017	0.919	578.999
	Hep-G2	25	1814.403	0.009	0.949	435.577	−0.003	0.609	737.681
		50	1691.575	0.010	0.922	533.418	0.010	0.957	622.296
		75	1441.026	0.009	0.902	538.309	0.020	0.928	585.877
		100	1048.643	0.008	0.869	1057.822	0.029	0.956	471.186
	Huh-7	25	2813.031	0.010	0.919	495.029	0.004	0.898	1034.856
		50	1957.116	0.008	0.903	365.556	−0.002	0.680	879.389
		75	1581.337	0.007	0.933	452.905	0.004	0.618	785.269
		100	1213.478	0.006	0.807	418.125	0.015	0.945	665.971
Bile	HCCC-9810	25	1719.363	0.007	0.870	322.897	0.003	0.268	853.810
		50	1691.929	0.010	0.920	358.120	0.003	0.419	622.426
		75	1611.441	0.015	0.921	563.781	0.005	0.774	359.561
		100	1466.889	0.016	0.931	697.996	0.012	0.887	296.160
	HIBEpiC	25	2883.189	0.010	0.955	570.890	−0.004	0.906	1060.666
		50	3153.721	0.013	0.994	695.075	−0.002	0.564	859.489
		75	2003.759	0.011	0.980	482.750	−0.005	0.730	666.993
		100	1866.801	0.019	0.979	629.820	0.005	0.469	279.215
	HuCCT-1	25	2592.384	0.011	0.982	775.667	0.006	0.953	862.930
		50	2403.929	0.012	0.959	784.966	0.007	0.998	724.050
		75	2292.476	0.013	0.968	853.363	0.008	0.800	624.773
		100	2175.557	0.015	0.945	845.391	0.011	0.891	485.432
	QBC-939	25	3297.848	0.012	0.987	1118.228	−0.001	0.178	993.293
		50	2671.958	0.018	0.945	893.800	0.008	0.882	441.672
		75	2157.099	0.018	0.979	923.800	0.007	0.786	356.566
		100	1923.263	0.024	0.963	967.059	0.008	0.716	174.474
Pancreas	MIA PaCa-2	25	2189.830	0.005	0.882	656.004	0.001	0.138	1328.199
		50	1735.254	0.007	0.848	538.921	0.005	0.695	861.702
		75	1488.322	0.007	0.759	528.346	0.009	0.839	739.079
		100	1300.553	0.006	0.848	569.525	0.012	0.888	713.759
	PANC-1	25**	NA	NA	NA	NA	NA	NA	NA
		50	1943.466	0.010	0.985	628.851	0.008	0.985	714.961
		75	1658.539	0.010	0.940	698.783	0.013	0.960	610.142
		100	1599.016	0.011	0.913	422.316	0.006	0.839	532.266

* E_*c*_(100) is the value of E_*c*_ under 100 pulses computed based on the calculated coefficient.

** The coefficient of Panc-1 cell line under 25 μs was calculated by modified Peleg-Fermi model and presented in Supplementary Table 2.

For the 25 μs pulse duration, most of the *E*_*c*_*(100)* values were in the range of 800–1,000 V/cm, and for a pulse duration of 100 μs, most of the *E*_*c*_*(100)* values were between 400 and 600 V/cm. For most of the cell lines, the value of *E*_*c*_*(100)* decreased with increasing pulse duration. When treated with EPs with a 100 μs pulse duration, the *E*_*c*_*(100)* value varied from 459 to 684 V/cm for esophageal cells, 85 to 536 V/cm for gastric cells, 316 to 632 V/cm for colon cells, 401 to 665 V/cm for hepatic cells, 174 to 485 V/cm for biliary cells, and 532 to 713 V/cm for pancreatic cells.

**Figure 3:**
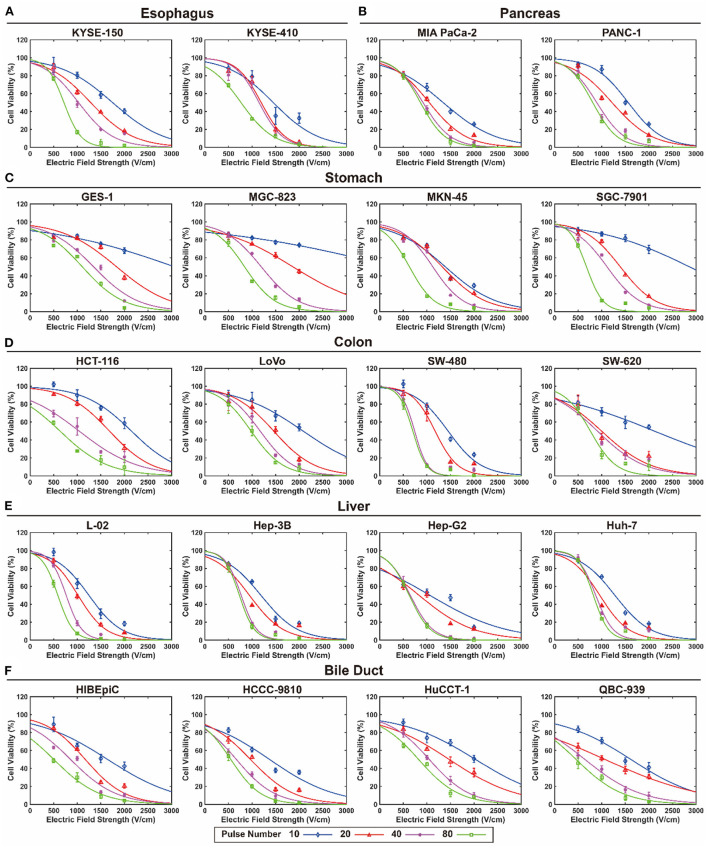
Survival curve of each cell line exposed to electroporation pulses with different pulse number and electric fields strength under 100 μs pulse duration. **(A)** Esophagus, **(B)** pancreas, **(C)** stomach, **(D)** colon, **(E)** liver, and **(F)** bile duct.

Figure 3 shows the calculated survival curve of each cell line exposed to EPs with different pulse numbers and EFS under a 100 μs pulse duration. The survival curves for pulse durations of 25, 50, and 75 μs are available in the online database (29).

### Evaluation of the model efficacy by numerical analysis

**Figure 4:**
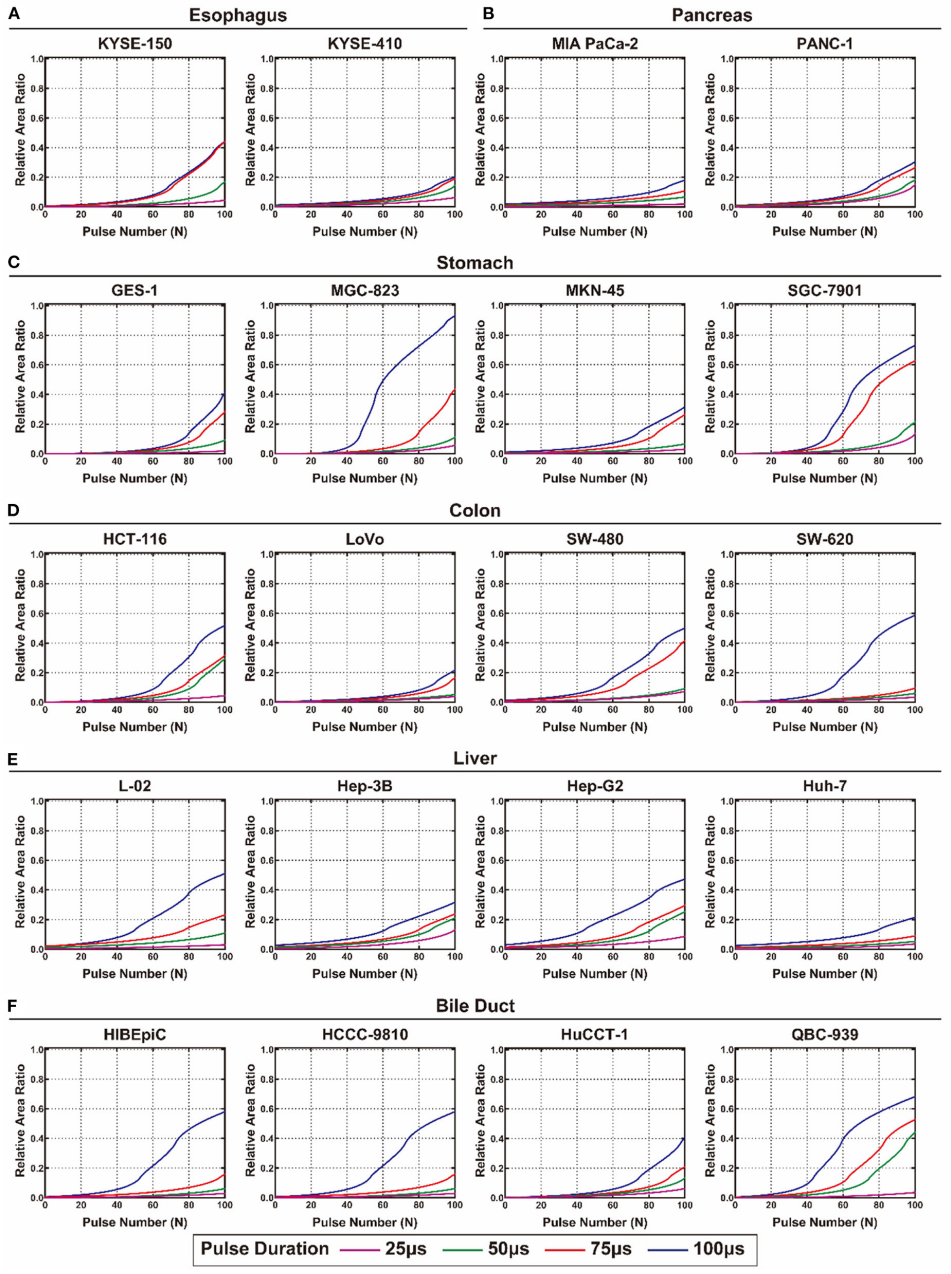
The calculated relative area ratio of the electrical injury to the digestive system. **(A)** Esophagus, **(B)** pancreas, **(C)** stomach, **(D)** colon, **(E)** liver, and **(F)** bile duct.

**Figure 5:**
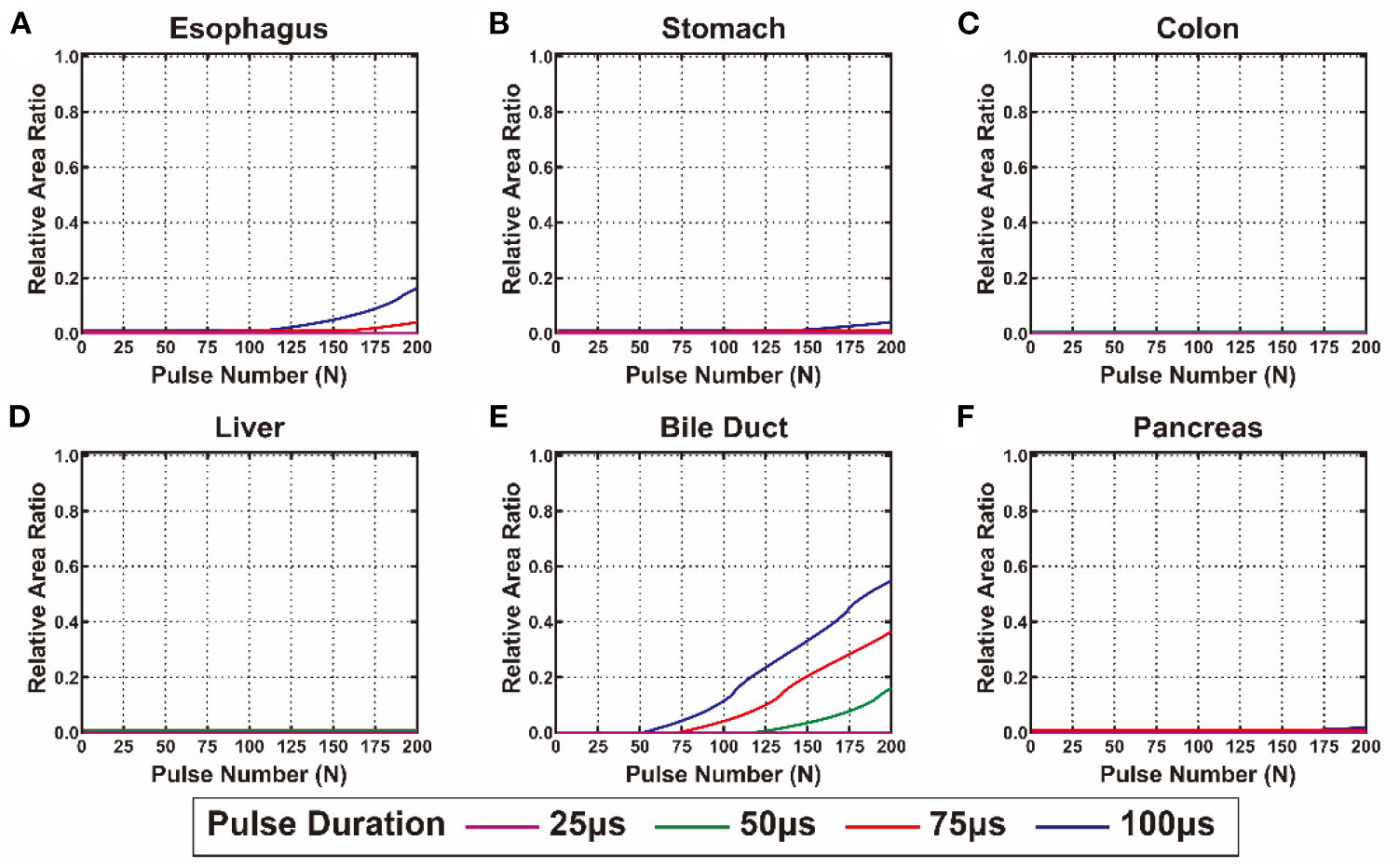
The calculated relative area ratio of the thermal injury to the digestive system. **(A)** Esophagus, **(B)** pancreas, **(C)** stomach, **(D)** colon, **(E)** liver, and **(F)** bile duct.

The calculated *S*_*EI*_ and *S*_*TI*_ of each cell line from the digestive system are shown in Figures 4, 5, respectively. The detailed values of *S*_*EI*_ and *S*_*TI*_ for specific pulse numbers can be found in the online database (29). All the cells showed a similar tendency: *S*_*EI*_ increased with the accumulation of pulse number and extension of pulse duration. The growth rate of *the S*_*EI*_ increased with increasing pulse duration; therefore, the *S*_*EI*_ with a pulse duration of 100 μs was the highest. The gastric cell lines (MGC-823 and SGC-7901) and cholangiocarcinoma cell lines (HCCC-9810 and QBC-939) showed higher slopes and maximum values of *the S*_*EI*_ curve than the other cell lines. In contrast, the viability of cell lines from the esophagus and pancreas showed a relatively lower inhibition rate when exposed to EPs (Figure 4).

In terms of TI, there was no substantial TI in the liver and colon when exposed to <200 pulses. For the esophagus, stomach, and pancreas, only slight TI was caused by EPs under the condition of 100 and 75 μs pulse duration. The bile duct is the most heat-sensitive site in the digestive system that presented with high *S*_*TI*_ under the conditions of 50, 75, and 100 μs pulse duration (Figure 5). Supplementary Table 3 summarizes the pulse number required for the occurrence and maximum value of EI and TI of each cell line. For all cell lines, more than 100 pulses were required to reach the maximal *S*_*EI*_ that would cause no TI in the colon and liver and mild TI in the stomach and pancreas. However, under these conditions, TI will occur in the esophagus treated by EPs with 100 μs pulse duration and in the bile duct treated by EPs with 50, 75, and 100 μs pulse duration.

## Discussion

In this study, a mathematical model database of cell survival after exposure to EPs from 20 types of human digestive system cell lines was established by *in vitro* and *in silico* studies. Moreover, the efficacy of the mathematical model for EBT was evaluated by numerical analysis for both EIs and TIs. This mathematical model database has potential value for theoretical analysis, computational simulation, numerical analysis, and treatment planning to help decision-making for basic research, medical device design, and clinical practice.

The cell survival model showed that cell viability decreased with an increase in pulse voltage, duration, and number, suggesting that the outcome of EPs is closely associated with the total electrical energy of EPs. This finding is consistent with that of Zheng et al., who showed IRE can cause substantial ablation volume in a 3D liver tumor model of Hep-G2 cells (30). In addition, Qi et al. have found that increased pulse number and higher electric field led to lower viability of both murine and human pancreatic cancer cells after IRE, and demonstrated that different cells have different susceptibility to IRE, suggesting the need for careful characterization of IRE response in the given cancer or tissue of interest (31). In this study, it is identical for most of the cell lines that the cell viability was not significantly affected by EPs under low EFS (<1,000 V/cm), and the EPs even exerted a positive effect on cell proliferation. When the EFS was >1,000 V/cm, cell viability was significantly decreased and was lower than 50%, implying that the EPs had a prominent negative effect on cell survival. This result was consistent with that of previous studies that the threshold for RE was lower than 500–800 V/cm in general, and the threshold for IRE was >1,000 V/cm (32). Therefore, selecting the appropriate pulse settings for a specific purpose is critical to the outcome of EBT. Notably, a low EFS promotes cell proliferation as the low intensity of electrical stimulation can promote adaptive proliferation of cancer cells (33).

For tissue ablation, there is general agreement that the EFS should be >1,000 V/cm to achieve IRE (34). However, the lethal effect of EPs is non-linear and cell-specific, implying that cell death due to EPs is an all-or-none phenomenon, and the threshold for electroporation differs between different cells. There is a complex relationship between pulse settings, cell types, and outcomes of cell viability. There are many factors affecting the outcomes of EPs, such as the cell shape, physical and chemical environment, composition of cell membrane, cholesterol content on cell membrane, cytoskeletal structural integrity, cell stiffness, cell volume, and transmembrane voltage (35). The lethal effect of EPs is more than a dose-response relationship because complete cell death relies on the cumulative effect of pulse voltage, duration, and number; thus, it is impossible to completely predict the outcome of EPs using simple pulse settings (32). Therefore, it is necessary to establish a mathematical model to comprehensively characterize the cell electroporation process.

The discrepancy between cancer and normal cells exposed to EPs is a problem worthy of attention (36). In a previous study, Danijela et al. found a disparate response to EPs between cancer and healthy cells (37). They demonstrated that cancer cells are more sensitive to EPs than normal cells, whereas in our study, the current data evidence cannot fully support this theory because there is a lack of an acceptable mathematical indicator to measure the EP sensitivity of a cell line. Nevertheless, the consensus is that cell sensitivity to EPs is determined by multiple factors, and it is possible to adjust the cell environment to promote the killing effect of EPs on cancer cells (35). Therefore, it is necessary to further investigate the mechanism of cell death by electroporation.

The Peleg–Fermi model was first introduced to microbial survival after exposure to PEFs by Peleg (11). This model contains two major factors of pulse settings: EFS and pulse number that can predict the cell survival rate under a specific pulse setting. Golberg et al. compared the performance of several mathematical models for EBT and demonstrated the value of the Peleg–Fermi model for prediction (27). There were two cell-specific coefficients, *E*_*c*_ and *A*_*c*_ that presented the change tendency of cell viability when exposed to EPs at the mathematical level. The *E*_*c*_ value showed a downward trend with the increase in EFS and pulse number, indicating that the EFS required for half lethality of the cells decreased when treated with EPs with enhanced energy. The *A*_*c*_ value showed a similar tendency with *E*_*c*_, indicating that the decline rate of cell viability caused by EPs was elevated with an increase in EFS and pulse number. The decline in *E*_*c*_ and *A*_*c*_ suggests an elevation of cell death exposed to EPs, which can be deduced from the Peleg–Fermi equation and the results above. In clinical practice, multiple series of EP bursts were performed on tissues for IRE. A total of 100 pulses were commonly set in a single burst of EPs; therefore, the *E*_*c*_*(100)* of all cell lines was calculated and compared in this study. For most cell lines, the value of *E*_*c*_*(100)* was approximately 500–700 V/cm, which is generally considered as the threshold for electroporation (3).

However, the pulse duration and electric-thermal coupling effect have not been taken into consideration in the Peleg–Fermi model; therefore, the survival rate was only calculated for several given pulse durations, and TI was calculated independently using the Arrhenius equation. Thus, a more accurate and comprehensive model for electroporation needs to be established in future studies. Based on the Peleg–Fermi model, the probability of EI can be calculated for a series of EPs. However, the probability of TI should be considered during electroporation treatment because tissue resistance can induce Joule heating by electrical energy. Therefore, the heat sensitivity of the digestive system was calculated and compared. In this study, there were significant differences between the digestive organs with respect to TI. For example, no TI occurred in the colon and liver, mild TI occurred in the esophagus, stomach, and pancreas, whereas significant TI occurred in the bile duct under the same pulse settings. Thermal injury might be associated with tissue components, blood perfusion rate, and tissue metabolism.

Neven et al. performed IRE on the outer esophageal wall and showed evidence of cell death at 2 days (macroscopically visible lesion) and 60 days (microscopically visible scar). They also found that harmless, self-limiting adverse effect and lasting tissue regeneration occurred during the 60 days follow-up (38). Recently, Song et al. further validated the safety of pulsed field ablation on the esophagus in a rabbit model (39). Our previous studies have confirmed the safety and efficacy of gastric tissue IRE ablation (21, 22). However, the numerical analysis of IRE in the digestive tract was performed based on prostate cancer cells because of the lack of available data for digestive tumors (16). In addition, the bile duct has been treated as heat-sensitive; therefore, TI to the bile duct may result in complications such as bile leaks and bile duct strictures (40). In this study, relatively more TI occurred in the bile duct, suggesting that the electrical energy should be controlled when applied to the bile duct. In our previous study, biliary stricture was found after exposure of the bile duct to EPs in a rabbit model, which was consistent with the findings of a clinical report (24). Biliary stricture may be associated with fibrosis in the mucosal layer, which is easily induced by heating. Our results indicated the necessity of avoiding any risk of collateral injury and precisely determining the pulse settings. Therefore, a numerical analysis is needed for heat-sensitive organs when performing EBT. Thus, herein we provide a survival model database of cell-specific coefficients based on the Peleg–Fermi model for the whole digestive system, which can be used for further application of EBT.

This study has some limitations. First, the cell survival model was established based on *in vitro* cell experiments that cannot completely reflect the effect of EPs at the tissue level. However, there is still no qualified method for directly evaluating cell activity under EP *in vivo*. This study can assess the biological effect of EPs on the survival of the digestive system to a large extent, laying a theoretical foundation for future studies. Second, the Peleg–Fermi model contains the EFS, pulse number, and pulse duration without frequency; however, pulse frequency may have a considerable effect on both EIs and TIs. This model may not be suitable for high-frequency EPs. Third, the EI and TI were calculated via the Peleg–Fermi equation and Arrhenius equation; however, there may exist an electrical-thermal coupling effect during electroporation, which needs to be further investigated for a more accurate model. Additionally, normal tissue properties were used to establish the numerical analysis model due to the lack of data from the tumor; therefore, the efficacy of this model needs to be further verified by *in vivo* studies in the future.

## Conclusions

In this study, a mathematical model database for cell survival containing cell-specific coefficients of 20 human digestive cell lines was established based on *in vitro* cell experiments and *in silico* analysis. The efficacy of the database was evaluated by numerical analysis of both EIs and TIs of the digestive system. This database can be used for basic research, computational simulation, medical device design, and treatment planning that can predict cell survival and tissue injury distribution after exposure to EPs during EBT.

## Data availability statement

The original contributions presented in the study are included in the article/Supplementary materials, further inquiries can be directed to the corresponding authors.

## Author contributions

XH and NZ designed the study, performed the related experiment, and wrote and edited the manuscript. YZ, ZL, JZ, XC, and ZQ researched and analyzed the data, helped in writing, and reviewed the manuscript. QL, YW, LM, TH, and YLi helped to perform the related experiments. FR and YLv designed the study and revised the manuscript. All authors have approved the final manuscript.

## Funding

This study was supported by National Natural Science Foundation of China (grant no. 81727802), the Fundamental Research Funds for Central Universities (grant no. XJH012020022), the Scientific Development Funding of the First Affiliated Hospital of Xi'an Jiaotong University (2020QN-08), and the Basic Natural Science Research Project of Shaanxi Province (2021JQ-401). The funders had no role in the design of the study and collection, analysis, and interpretation of data and in writing the manuscript.

## Conflict of interest

The authors declare that the research was conducted in the absence of any commercial or financial relationships that could be construed as a potential conflict of interest.

## Publisher's note

All claims expressed in this article are solely those of the authors and do not necessarily represent those of their affiliated organizations, or those of the publisher, the editors and the reviewers. Any product that may be evaluated in this article, or claim that may be made by its manufacturer, is not guaranteed or endorsed by the publisher.

## Abbreviations

EP: electroporation pulse
EFS: electric field strength
EBT: electroporation-based treatment
RE: reversible electroporation
IRE: irreversible electroporation
EI: electrical injury
TI: thermal injury.
