# Supplementary material for: Survival model database of human digestive system cells exposed to electroporation pulses: An in vitro and in silico study
Source: Front Public Health. 2022 Sep 5;10:948562. doi: 10.3389/fpubh.2022.948562 (PMC9484541; doi:10.3389/fpubh.2022.948562)
Supplement: Supplementary file 1 [file Data_Sheet_1.docx]

**Supplementary Tables**

**Supplementary Table 1.** Parameter setup protocol for EP treatment

| Voltage (*V*) | Electric field(*V/cm*) | Pulse width(*μs*) | Pulse number(*N*) |
| --- | --- | --- | --- |
| 100 | 500 | 25 | 10, 20, 40, 80 |
|  |  | 50 | 10, 20, 40, 80 |
|  |  | 75 | 10, 20, 40, 80 |
|  |  | 100 | 10, 20, 40, 80 |
| 200 | 1000 | 25 | 10, 20, 40, 80 |
|  |  | 50 | 10, 20, 40, 80 |
|  |  | 75 | 10, 20, 40, 80 |
|  |  | 100 | 10, 20, 40, 80 |
| 300 | 1500 | 25 | 10, 20, 40, 80 |
|  |  | 50 | 10, 20, 40, 80 |
|  |  | 75 | 10, 20, 40, 80 |
|  |  | 100 | 10, 20, 40, 80 |
| 400 | 2000 | 25 | 10, 20, 40, 80 |
|  |  | 50 | 10, 20, 40, 80 |
|  |  | 75 | 10, 20, 40, 80 |
|  |  | 100 | 10, 20, 40, 80 |

**Supplementary Table 2.** The coefficient of Panc-1 cell line under 25μs

| Organ | Cell line | Pulse Duration(μs) | *E_1_* (V/cm) | *k_1_* | *E_2_* (V/cm) | *k_2_* | *A_1_* (V/cm) | *k_3_* | *A_2_* (V/cm) | *k_4_* | *E_c_(100)*(V/cm)***** |
| --- | --- | --- | --- | --- | --- | --- | --- | --- | --- | --- | --- |
| Pancreas | PANC-1 | 25 | 3885.000 | 7.292 | 2373.505 | 0.012 | 405.287 | -0.002 | 3494.908 | 0.201 | 690.180 |

******E_c_(100)* is the value of *E_c_* under 100 pulses computed based on the calculated coefficient.

**Supplementary Table 3.** The pulse number required for the occurrence and maximum of EI and TI of each cell line.

| Organ | Cell line | Electrical Injury | | | | Thermal Injury | | | |
| --- | --- | --- | --- | --- | --- | --- | --- | --- | --- |
|  |  | Maximum | | | | Occurrence | | | |
|  |  | 25μs | 50μs | 75μs | 100μs | 25μs | 50μs | 75μs | 100μs |
| Esophagus | KYSE-150 | 106 | 104 | 103 | 103 | >200 | >200 | 138 | 93 |
|  | KYSE-410 | 105 | 102 | 104 | 107 |  |  |  |  |
| Stomach | GES-1 | 108 | 105 | 102 | 102 | >200 | >200 | 182 | 119 |
|  | MGC-823 | 105 | 104 | 104 | 101 |  |  |  |  |
|  | MKN-45 | 104 | 103 | 103 | 105 |  |  |  |  |
|  | SGC-7901 | 103 | 103 | 102 | 101 |  |  |  |  |
| Colon | HCT-116 | 106 | 107 | 114 | 113 | >200 | >200 | >200 | >200 |
|  | LoVo | 103 | 103 | 102 | 104 |  |  |  |  |
|  | SW-480 | 106 | 107 | 106 | 102 |  |  |  |  |
|  | SW-620 | 105 | 109 | 109 | 102 |  |  |  |  |
| Liver | L-02 | 107 | 106 | 103 | 103 | >200 | >200 | >200 | >200 |
|  | Hep-3B | 105 | 104 | 103 | 103 |  |  |  |  |
|  | Hep-G2 | 114 | 105 | 102 | 102 |  |  |  |  |
|  | HuH-7 | 105 | 109 | 110 | 104 |  |  |  |  |
| Bile duct | HIBEpiC | 112 | 112 | 117 | 111 | >200 | 104 | 63 | 44 |
|  | HCCC-9810 | 106 | 106 | 109 | 107 |  |  |  |  |
|  | HuCCT-1 | 107 | 106 | 107 | 106 |  |  |  |  |
|  | QBC-939 | 117 | 107 | 110 | 115 |  |  |  |  |
| Pancreas | MIA PaCa-2 | 113 | 108 | 106 | 105 | >200 | >200 | >200 | 134 |
|  | PANC-1 | 109 | 106 | 105 | 106 |  |  |  |  |

**Supplementary Materials and Methods**

1. **Numerical Analysis**

***1.1 Models of tissue and electrode***

The electrode with radius of 1 mm was set at the middle of the rectangle tumor tissue.


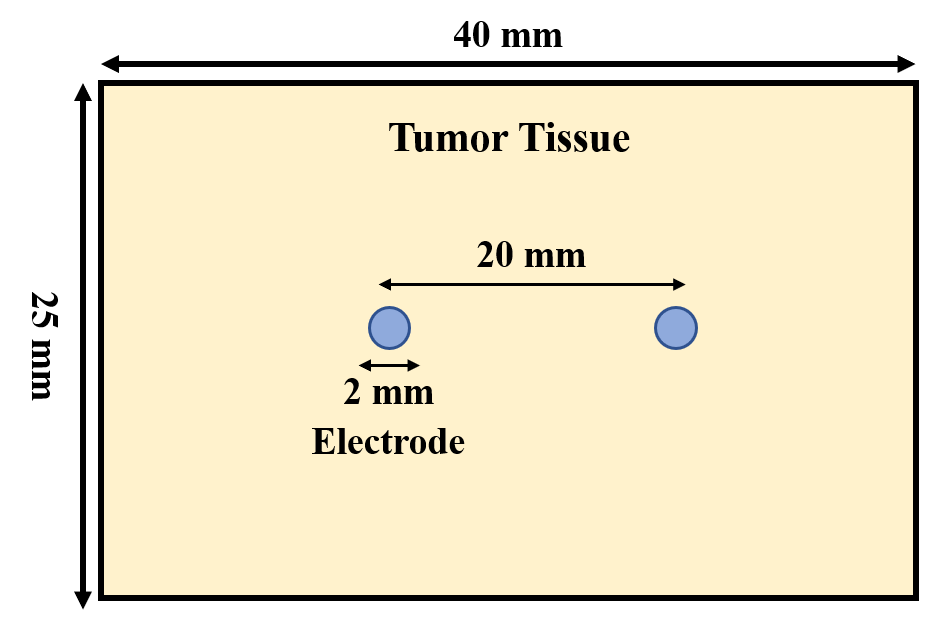


**Figure 1.** Two-dimensional model of tumor tissue and needle-electrodes

***1.2 Calculation of electric field distribution***

The electric filed distribution was determined by Laplace equation:

$\nabla\cdot\left( \sigma\nabla\varphi\right)=0$ (1)

where *σ* is the electrical conductivity of the tissue, and *φ* is the electrical potential at a specific location.

- 1. ***Calculation of temperature distribution***

The temperature distribution was determined by modified Pennes bioheat transfer equation:

$\nabla\cdot\left( k\nabla T \right)+\omega_{b}c_{b}\left( T_{a}-T \right)+Q_{m}+Q_{Joule}=\rho c_{t}\frac{\partial T}{\partial t}$ (2)

$Q_{Joule}=\sigma\left| \nabla\varphi\right|^{2}$ (3)

where *k* is the thermal conductivity, *T* is the temperature, *ω_b_* is the blood perfusion rate, *c_b_* is the heat capacity of the blood, *T_a_* is the arterial temperature, *Q*_m_ is the metabolic heat generation of the tissue, *Q_Joule_* is the Joule heating accounts for resistive heating of tissue, *ρ* is the tissue density, and *c_t_* is the heat capacity of tissue.

- 1. ***Calculation of cell death probability during IRE***

The cell death probability during IRE was determined by Peleg-Fermi model:

$S=\frac{1}{1+e^{\frac{E-E_{c}\left( N \right)}{A_{c}\left( N \right)}}}$ (4)

$E_{c}\left( N \right)=E_{0}\exp\left( -k_{1}n \right)$ (5)

$A_{c}\left( N \right)=A_{0}\exp\left( -k_{2}n \right)$ (6)

where *S* is the proportion of surviving cells after IRE and *E* is the electrical field strength. *E_c_(N)* and *A_c_(N)* are two functions that depend on the number of pulses. The *E_0_, A_0_, k_1_* and *k_2_* are the regression coefficients and are related to the pulse duration and cell type.

$E_{c}\left( N \right)=E_{c1}exp \left( -k_{1}N \right)+E_{c2}exp \left( -k_{2}N \right)$ (7)

$A_{c}\left( N \right)=A_{1}exp \left( -k_{3}N \right)+A_{2}\exp\left( -k_{4}N \right)$ (8)

where *E_1_, E_2_, A_1_, A_2_, k_1_, k_2_, k_3_, and k_4_* are the coefficients related to the pulse duration, frequency, and cell type.

The probability of EI can be calculated as:

$Probability of EI \left( PEI \right)=1-S$ (9)

- 1. ***Calculation of TI probability during IRE***

The TI probability during IRE was determined by Arrhenius rate equation [1], [2]:

$\Omega\left( t \right)=\int_{t=0}^{t=\tau} \xi exp\left( \frac{-E_{a}}{RT\left( t \right)} \right)dt$ (10)

where *R* is the universal gas constant (8.314 J/(mol·K)), *ξ* is the frequency factor (7.39×10^39^ 1/s), *E_a_* is the activation energy (2.577×10^5^ J/mol), *T*(*t*) is the temperature distribution at a specific time, and *τ* is the heating time. The temperature distribution was calculated using Equation 2. The frequency factor and activation energy are tissue-specific parameters that describe different modes of TI [3], [4]. The probability of cell death due to TI can be calculated as follows:

$Probability of TI \left( PTI \right)= 1-\exp\left( -\Omega\left( t \right) \right)$ (11)

- 1. ***Boundary and initial conditions***

The electrode–tissue interface was defined as thermally continuous and be applied by the Dirichlet boundary condition. The external boundary of the tissue-electrode was set as adiabatic and insulative.

The left electrode was set as anode with a potential of 2000V, while the right electrode was set as cathode with a potential of 0V. The initial temperature of the tissue and artery was set to 37 ℃.

$\varphi_{anode}=2000$ (12)

$\varphi_{cathode}=0$ (13)

**Reference:**

[1] P. A. Garcia, R. V. Davalos, and D. Miklavcic, “A numerical investigation of the electric and thermal cell kill distributions in electroporation-based therapies in tissue,” PLoS One, vol. 9, no. 8, 2014, doi: 10.1371/journal.pone.0103083.

[2] H. Shafiee, P. A. Garcia, and R. V Davalos, “A Preliminary Study to Delineate Irreversible Electroporation From Thermal Damage Using the Arrhenius Equation,” J. Biomech. Eng., vol. 131, no. 7, Jun. 2009, doi: 10.1115/1.3143027.

[3] M. J. Borrelli, L. L. Thompson, C. A. Cain, and W. C. Dewey, “Time-temperature analysis of cell killing of BHK cells heated at temperatures in the range of 43.5°C to 57.0°C,” Int. J. Radiat. Oncol., vol. 19, no. 2, pp. 389–399, 1990, doi: https://doi.org/10.1016/0360-3016(90)90548-X.

[4] S. L. Brown, J. W. Hunt, and R. P. Hill, “Differential thermal sensitivity of tumour and normal tissue microvascular response during hyperthermia,” Int. J. Hyperth., vol. 8, no. 4, pp. 501–514, Jan. 1992, doi: 10.3109/02656739209037988.

1. **MATLAB program code**

2.1 %Cell Viability Plotting

Edata=[500 1000 1500 2000]; %Electric field strength input, the same below.

Sdata=xlsread('Cell-survival.xlsx','Sheet2','C4:F4'); %Cell survival data input, the same below.

plot(Edata,Sdata,'-db','LineWidth',2,'MarkerSize',8);

hold on

err=xlsread('Cell-survival.xlsx','Sheet2','C5:F5'); %Standard deviation data input, the same below.

errorbar(Edata,Sdata,err,'bd','MarkerSize',8,'LineWidth',2);

Sdata=xlsread('Cell-survival.xlsx','Sheet2','C8:F8');

plot(Edata,Sdata,'-^r','LineWidth',2,'MarkerSize',8)

hold on

err=xlsread('Cell-survival.xlsx','Sheet2','C9:F9');

errorbar(Edata,Sdata,err,'r^','MarkerSize',8,'LineWidth',2);

Sdata=xlsread('Cell-survival.xlsx','Sheet2','C12:F12');

plot(Edata,Sdata,'-*m','LineWidth',2,'MarkerSize',8);

hold on

err=xlsread('Cell-survival.xlsx','Sheet2','C13:F13');

errorbar(Edata,Sdata,err,'m*','MarkerSize',8,'LineWidth',2);

Sdata=xlsread('Cell-survival.xlsx','Sheet2','C16:F16');

plot(Edata,Sdata,'-sg','LineWidth',2,'MarkerSize',8);

hold on

err=xlsread('Cell-survival.xlsx','Sheet2','C17:F17');

errorbar(Edata,Sdata,err,'gs','MarkerSize',8,'LineWidth',2);

xlim([0 2500])

ylim([0,120])

set(gca,'FontName','Arial','FontWeight','bold','FontSize',16,'LineWidth',2)

xlabel('Electric Field Strength (V/cm)','FontSize',18,'FontName','Arial')

ylabel('Cell Viability (%)','FontSize',18,'FontName','Arial')

title('Pulse Duration = 25μs','FontName','Arial','FontWeight','bold','FontSize',20)

print(gcf,'25','-dtiff','-r1200')

clf

clear

clc

2.2 %Curve Fitting and Plotting of Peleg-Fermi model (Ec and Ac)

%Curvefun1:

function f=curvefun1(x,Edata)

f=100./(1+exp((Edata-x(1))./x(2)));%x(1)=Ec, x(2)=Ac

Edata=[500 1000 1500 2000]; %Electric field strength input.

Sdata=xlsread('Cell-survival.xlsx','Sheet2','C4:F4'); %Cell survival data input.

x0=[100 100]; %Note: x0 is the initial value of curve fitting which was set empirically here and might need modified for specific data, however, it causes little impact on the results in most cases.

x=lsqcurvefit('curvefun1',x0,Edata,Sdata); %Ec and Ac value calculation.

f=curvefun1(x,Edata);

x1=linspace(0,3000,500);

plot(x1, curvefun1 (x,x1), 'b-','LineWidth',2);

hold on

err=xlsread('Cell-survival.xlsx','Sheet2','C5:F5'); %Standard deviation data input.

errorbar(Edata,Sdata,err,'bd','MarkerSize',8,'LineWidth',2)

R1= corrcoef(Sdata, f); %R square calculation.

R2=R1(2,1);

xlswrite('Cell-survival.xlsx',x,'Sheet3','C2:D2') %Ec and Ac value output.

xlswrite('Cell-survival.xlsx',R2,'Sheet3','E2') %R square output.

axis([0 3000 0 120])

set(gca,'FontName','Arial','FontWeight','bold','FontSize',16,'LineWidth',2)

xlabel('Electric Field Strength (V/cm)','FontSize',18,'FontName','Arial')

ylabel('Cell Viability (%)','FontSize',18,'FontName','Arial')

title('Pulse Duration = 25μs','FontName','Arial','FontWeight','bold','FontSize',20)

print(gcf,'25-fit','-dtiff','-r1200')

clf

2.3 %Curve Fitting and plotting of Peleg-Fermi model (E0, k1, A0 and k2)

%Curvefun4:

function f=curvefun4(x,ndata)

f=x(1)* exp (-x(2)*ndata); %x(1)=E0, x(2)=k1

%Curvefun5:

function f=curvefun5(x,ndata)

f=x(1)* exp (-x(2)*ndata); %x(1)=A0, x(2)=k2

Edata=[10 20 40 80]; %Pulse number input, the same below.

Sdatarow=xlsread('Cell-survival.xlsx','Sheet3','C2:C5'); %Ec value input.

Sdata=Sdatarow';

x0=[3885 -0.6]; %Note: x0 is the initial value of curve fitting which was set empirically here and might need modified for specific data, however, it causes little impact on the results in most cases. The same below.

x=lsqcurvefit('curvefun4',x0,Edata,Sdata); % E0 and k1 calculation.

f=curvefun4(x,Edata);

x1=linspace(0,100,1000);

hold on

plot(Edata,Sdata, 'ok',x1, curvefun4 (x,x1),'-k','LineWidth',2,'MarkerSize',8)

R1= corrcoef(Sdata, f); %R square calculation, the same below.

R2=R1(2,1);

xlswrite('Cell-survival.xlsx',x,'Sheet4','C2:D2') % E0 and k1 value output.

xlswrite('Cell-survival.xlsx',R2,'Sheet4','E2') %R square output, the same below.

Edata=[10 20 40 80];

Sdatarow=xlsread('Cell-survival.xlsx','Sheet3','D2:D5'); %Ac value input.

Sdata=Sdatarow';

x0=[40 0.02] ;

x=lsqcurvefit('curvefun5',x0,Edata,Sdata); % A0 and k2 value calculation.

f=curvefun5(x,Edata);

x1=linspace(0,100,1000);

hold on

plot(Edata,Sdata, '*r',x1, curvefun5 (x,x1),'--r','LineWidth',2,'MarkerSize',8)

R1= corrcoef(Sdata, f);

R2=R1(2,1);

xlswrite('Cell-survival.xlsx',x,'Sheet5','C2:D2') % A0 and k2 value output.

xlswrite('Cell-survival.xlsx',R2,'Sheet5','E2')

set(gca,'YLim',[0 6000], 'XLim',[0 100],'FontName','Arial','FontWeight','bold','FontSize',16,'LineWidth',2)

xlabel('Pulse Number','FontSize',18,'FontName','Arial')

ylabel('Electric Field Strength (V/cm)','FontSize',18,'FontName','Arial')

title('Pulse Duration = 25μs','FontName','Arial','FontWeight','bold','FontSize',20)

box on

print(gcf,'25-basic','-dtiff','-r1200')

clf

2.4 %Curve Fitting of Modified Peleg-Fermi model (E1, E2, A1, A2, k1, k2, k3, and k4)

%Curvefun2:

function f=curvefun2(x,ndata)

f=x(1)* exp (-x(2)*ndata)+x(3)*exp(-x(4)*ndata); %x(1)=E1, x(2)=k1, x(3)=E2, x(4)=k2

%Curvefun3:

function f=curvefun3(x,ndata)

f=x(1)* exp (-x(2)*ndata)+x(3)*exp(-x(4)*ndata); %x(1)=A1, x(2)=k3, x(3)=A2, x(4)=k4

Edata=[10 20 40 80]; %Pulse number input, the same below.

Sdatarow=xlsread('Cell-survival.xlsx','Sheet3','C2:C5'); %Ec value input.

Sdata=Sdatarow';

x0=[3885 0.01 2.3 -0.6];

x=lsqcurvefit('curvefun2',x0,Edata,Sdata); %E1, E2, k1 and k2 calculation.

f=curvefun2(x,Edata);

x1=linspace(0,100,1000);

hold on

plot(Edata,Sdata, 'ok',x1, curvefun2 (x,x1),'-k','LineWidth',2,'MarkerSize',8)

R1= corrcoef(Sdata, f); %R square calculation, the same below.

R2=R1(2,1);

xlswrite('Cell-survival.xlsx',x,'Sheet6','B2:E2') %E1, E2, k1 and k2 output.

xlswrite('Cell-survival.xlsx',R2,'Sheet6','F2') %R square output, the same below.

Edata=[10 20 40 80];

Sdatarow=xlsread('Cell-survival.xlsx','Sheet3','D2:D5'); %Ac value input.

Sdata=Sdatarow';

x0=[40 -0.03 902 0.02];

x=lsqcurvefit('curvefun3',x0,Edata,Sdata); %A1, A2, k3, and k4 calculation.

f=curvefun3(x,Edata);

x1=linspace(0,100,1000);

hold on

plot(Edata,Sdata, '*r',x1, curvefun3 (x,x1),'--r','LineWidth',2,'MarkerSize',8)

R1= corrcoef(Sdata, f);

R2=R1(2,1);

xlswrite('Cell-survival.xlsx',x,'Sheet7','B2:E2') %A1, A2, k3, and k4 output.

xlswrite('Cell-survival.xlsx',R2,'Sheet7','F2')

set(gca,'YLim',[0 6000], 'XLim',[0 100],'FontName','Arial','FontWeight','bold','FontSize',16,'LineWidth',2)

xlabel('Pulse Number','FontSize',18,'FontName','Arial')

ylabel('Electric Field Strength (V/cm)','FontSize',18,'FontName','Arial')

title('Pulse Duration = 25μs','FontName','Arial','FontWeight','bold','FontSize',20)

box on

print(gcf,'25-improved','-dtiff','-r1200')

clf

2.5 %Curve Fitting of power function model (a, b)

%Curvefun6:

function f=curvefun6(x,tp)

f=x(1) * (tp/1) .^x(2); %x(1)=a,x(2)=b

tp=xlsread('Panc1.xlsx','Sheet1','B2:E2'); %pulse duration input.

E=xlsread('Panc1.xlsx','Sheet1','B3:E3'); %Electric filed strength input.

x0=[0 0]; %Note: x0 is the initial value of curve fitting which was set empirically here and might need modified for specific data.

x=lsqcurvefit('curvefun6',x0,tp,E); %a, b value calculation.

f=curvefun6(x,tp);

xlswrite('Panc1.xlsx',x,'Sheet1','G3:H3') %a, b value output.

R1= corrcoef(E, f); %R square calculation.

R2=R1(2,1);

xlswrite('Panc1.xlsx',R2,'Sheet1','I3') %R square output.
